# Exploring the Psoriatic Arthritis Proteome in Search of Novel Biomarkers

**DOI:** 10.3390/proteomes6010005

**Published:** 2018-01-24

**Authors:** Shalini M. Mahendran, Vinod Chandran

**Affiliations:** 1 Department of Laboratory Medicine and Pathobiology, University of Toronto, Toronto, ON M5S 1A1, Canada; m.mahendran@mail.utoronto.ca; 2 Department of Pathology and Laboratory Medicine, Mount Sinai Hospital, Toronto, ON M5T 3L9, Canada; 3 Centre for Prognosis Studies in Rheumatic Diseases, Krembil Research Institute, Toronto Western Hospital, University Health Network, Toronto, ON M5T 1M8, Canada; 4 Division of Rheumatology, Department of Medicine, University of Toronto, Toronto, ON M5T 1A1, Canada; 5 Institute of Medical Science, University of Toronto, Toronto, ON M5T 1A1, Canada

**Keywords:** proteomics, biomarkers, psoriatic arthritis, serum, synovial fluid

## Abstract

Psoriatic arthritis (PsA) is an inflammatory arthritis which develops in up to one-third of patients suffering from the cutaneous disorder, psoriasis. The complex and heterogeneous nature of PsA renders it difficult to diagnose, leading to poor outcomes and, therefore, warrants an examination into soluble biomarkers, which may facilitate early detection of the disease. Protein biomarkers are a dynamic resource of pathophysiological information able to provide an immediate reflection of pathological changes caused by disease. Investigations of the serum and synovial fluid of PsA patients has provided new insights into the molecular basis of this disease and led to the identification of sensitive diagnostic and prognostic biomarkers. The collection of novel PsA biomarkers identified through proteomic studies has been reviewed below.

## 1. Introduction

Psoriasis is a chronic, immune-driven cutaneous disease afflicting nearly 2% of the global population. Due to the hyperproliferative nature of the epidermis during the onset of psoriasis, individuals are typically distinguished by the development of scaly erythematous plaques on the elbows and knees, and less commonly, on the scalp, umbilicus and lumbar area [1]. Psoriasis is a strong risk factor for the development of several comorbidities of which the most common is psoriatic arthritis (PsA). PsA is an inflammatory arthritis that manifests in roughly 30% of psoriasis patients and in 0.25% of the population. The heterogeneous nature of the disease has contributed to its division into five clinical subtypes based on its phenotypic presentation. These subtypes include: oligoarticular, polyarticular, distal, axial, and arthritis mutilans [2].

Genetic, environmental and immunologic factors are believed to be some of the primary forces driving the complex pathophysiology of PsA. Familial aggregation studies have revealed a high recurrence risk of PsA among relatives of patients with several studies presenting a sibling recurrence risk ratio of more than 27 [3]. Class I major histocompatibility complex (MHC) alleles are thought to play an integral role in PsA development. Genetic association studies have identified HLA-B*08, HLA-B*27, HLA-B*38 and HLA-B*39 as strong risk factors for PsA development in psoriasis patients [4,5,6]. Conversely, the presence of HLA-C*06 has demonstrated protective effects in at-risk patients by delaying the onset of PsA [4]. Interrogation of the PsA genome using single-nucleotide polymorphisms (SNPs) has revealed additional genes likely contributing to disease onset. These include interleukin 23 receptor (IL-23R) and tumor necrosis factor alpha-induced protein 3 (TNFAIP3), which have been implicated in the development of PsA more so than in psoriasis [7]. Additionally, major histocompatibility complex class I chain-related A (MICA) alleles are also considered to be associated with PsA [8,9]. Taken together, genomic studies allude to a highly heritable genetic predisposition.

The polygenic nature of PsA contributes to the diverse range of clinical features which often impede its early detection and subsequent therapeutic intervention. Similarities to other inflammatory arthritides emphasize the need for a rigorous means of diagnosis during pre-symptomatic and early clinical stages to ensure prompt management of a potentially debilitating disease. Currently, PsA detection is guided by the criteria outlined in the Classification Criteria for Psoriatic Arthritis (CASPAR) [10]. This first includes a judgment by a rheumatologist that the patient has an inflammatory musculoskeletal disease, followed by a review of the individual’s family and personal history of psoriasis, past or present evidence of dactylitis, nail psoriasis, the absence of serum rheumatoid factor and radiographic evidence of new bone formation [2].

**Figure 1:**
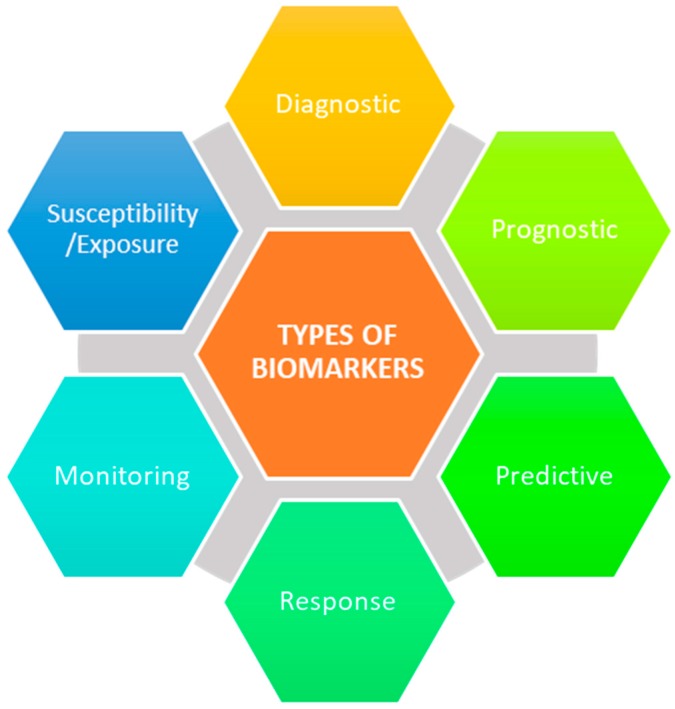
Biomarker categories.

The major roadblock in identifying PsA early is the lack of objective measures to detect musculoskeletal inflammation, especially in patients with cutaneous psoriasis (PsC) only. Consequently, researchers continue to probe for robust biomarkers of PsA, since traditional markers of inflammation have not been found to be particularly useful for diagnosis. A biomarker is defined by the National Institutes of Health Biomarkers Definitions Working Group as “a characteristic that is objectively measured and evaluated as an indicator of normal biological processes, pathogenic processes, or pharmacologic responses to therapeutic intervention” [11]. There are currently no clinically validated biomarker assays to detect the onset and activity of PsA. Among PsA patients, 95% test negative for rheumatoid factor and/or anti-cyclic citrullinated peptide antibodies, both of which are diagnostic biomarkers used for the detection of RA [2]. If positive, PsA patients often require additional clinical analysis including the use of imaging modalities, such as X-ray imaging or magnetic resonance imaging, to visualize the differences in radiographic progression of PsA and RA [12]. Additionally, once PsA has been confirmed in a patient, the use of specific biomarkers can resolve several unanswered questions, including whether the patient will experience a severe or mild form of the disease (prognostic), which therapy to initialize for the most optimal result (predictive), whether the disease is persisting or regressing (monitoring), and many more (Figure 1) [13]. Naturally, there is considerable interest in identifying novel biomarkers for PsA in PsC patients.

Proteins have served, and continue to serve, as a valuable source of physiological information. The inherent nature of the arthritis proteome confers a great advantage for the identification of novel markers by providing a real-time reflection of pathological changes occurring at the local and systemic level. The presence or absence of post-transcriptional and post-translational modifications (PTMs) necessary for protein functionality provides additional information regarding potentially aberrant mechanisms driving immuno-pathogenesis [14]. The dynamic state of protein levels and their respective PTMs aids in the ability to delineate key pathways involved in the onset of arthritic disease.

## 2. Serological Biomarkers 

The ease with which the serum can be accessed has rendered it an appealing source of putative proteomic biomarkers of PsA. Presently, serum levels of non-specific inflammatory marker C-reactive protein (CRP) serve as a measure of disease activity in PsA but also in several other inflammatory conditions. In search of better prognostic markers of PsA, Hansson et al. assayed serum S100 calcium-binding protein A8 (S100A8)/S100A9 (S-calprotectin) concentrations in 65 PsA with varying degrees of disease severity [15]. S-calprotectin levels were significantly higher in PsA patients with polyarthritis as opposed to mono-/oligoarthritis and, overall, were a better predictor for PsA than CRP levels. Their findings highlight the prognostic capabilities of S-calprotectin as a disease activity marker in PsA patients specifically. A detailed list of candidate PsA protein biomarkers described in this review can be found in Table 1. In-depth investigation of the PsA serum proteome has revealed the upregulation of several pro-inflammatory mediators, including cytokines and chemokines [16]. Alenius et al. detected significantly higher levels of interleukin-6 (IL-6) in PsA patients relative to PsC patients, regardless of their levels of routine measurable inflammatory markers [17]. IL-6 also correlated with increased levels of highly sensitive CRP (hs-CRP) and the erythrocyte sedimentation rate (ESR) among PsA patients with joint manifestations, among which there were higher incidences of polyarthritic disease pattern. Levels of IL-6 may, therefore, capture the disease activity of PsA more specifically than current parameters. The C–X–C motif chemokine 10 (CXCL10) has recently been identified as a potential predictor of PsA development among PsC patients [18]. Luminex assays of baseline serum concentrations of CXCL10 revealed a significantly higher level of the protein in PsC patients who later developed PsA by the annual follow-up visit. These levels however, were shown to decrease post-conversion for reasons that remain to be fully elucidated. More diagnostic biomarkers were discovered by ELISA when Chandran et al. quantified potential candidates in the serum of PsA patients [19]. Their findings confirmed increased levels of hsCRP, osteoprotegerin (OPG), matrix metalloproteinase 3 (MMP-3), as well as the ratio of C-propeptide Type II collagen to Col2-3/4C_long mono_ (CPII:C2C) in PsA patients compared to PsC patients. Moreover, they determined CD5-like antigen (CD5L), integrin subunit beta 5 (ITGB5), myeloperoxidase (MPO), Mac-2 binding protein (M2BP), MMP-3 and CRP were strong diagnostic markers of PsA and, when used in combination, they could differentiate PsA from PsC patients better than the use of CRP alone [20]. The search for circulating autoantibodies in PsA patients led Dalmady et al. to quantify antibodies targeting mutated citrullinated vimentin (anti-MCVs) by ELISA. They observed that PsA patients had significantly increased mean serum anti-MCV concentrations relative to PsC patients and emphasized the possibility of anti-MCVs to serve as a differentiator between mild and severe forms of PsA [21].

There is a growing interest in detecting early changes in serum biomarker concentrations in response to the initiation of drug therapy. Predictive biomarkers are crucial for distinguishing treatment responders and non-responders to ensure patients are receiving optimal therapy and to avoid unnecessary expenses and risk of adverse events. Several protein candidates have been identified as potential biomarkers of TNF-α inhibitor (TNFi) therapy response. Kuijk et al. investigated early changes in soluble biomarkers in response to initiation of adalimumab treatment [22]. They observed a significant decrease in serum MMP-3 levels and a significant increase in serum melanoma inhibitory activity (MIA) levels following 4 weeks of treatment. Results of a serum biomarker study by Chandran et al. similarly identified baseline MMP-3 reductions to be associated with response to TNFi therapy [23]. They also noted an independent association of increased serum cartilage oligomeric matrix protein (COMP) levels to TNFi therapy response. Cauza et al. also quantified the concentration of COMP in the serum of PsA patients following treatment with infliximab, a TNF-α blocker [24]. However, COMP levels were shown to significantly decrease after 6 weeks of treatment. The conflicting results between these two studies would suggest that COMP may not serve as a reliable marker for response to TNFi treatment. Alternatively, serum MMP-3 and MIA levels are more likely to assist in differentiating between effective and ineffective responses to TNF-α blockers.

High-throughput mass spectrometry (MS) analysis has opened novel avenues for extensive protein identification and quantification. When coupled with heparin affinity chromatography, a total of 384 proteins were identified among RA, PsA, and non-inflammatory arthritis plasma samples, of which 4 proteins were determined to be differentially regulated between RA and PsA. Serpin A11 (SERPINA11), complement factor H-related protein 5 (CFHR5), cartilage acidic protein 1 (CRTAC1) and coagulation factor IX (F9) were specifically upregulated in RA samples but downregulated in PsA samples, suggesting these proteins as a collective may accurately distinguish between PsA or RA patients [25].

## 3. Synovial Biomarkers

The synovial fluid (SF) is an ultrafiltrate of plasma and a reservoir of proteins shed from the synovial membrane, articular cartilage, and blood. Its dynamic proteome fluctuates in response to pathophysiological changes of the joint structure and, hence, is a coveted source of putative protein biomarkers of arthritic disease. Scrivo et al. explored the role of SF cytokines IL-19, IL-20, and IL-24 in sustaining the auto-immune/auto-inflammatory response of PsA [16]. IL-19 was observed to have unique elevation in PsA SF relative to matched serum and RA SF levels. This would suggest the involvement of IL-19 in a localized inflammatory response; however, its role in the progression of PsA has yet to be probed. Similarly, IL-9 concentrations were greater in PsA SF than in serum and it is postulated to be a driver of synovial T cell proliferation [29]. Collectively, this may implicate both IL-9 and IL-19 in the progression of PsA. Cytokines and chemokines have also been investigated as pharmacodynamic biomarkers in response to intra-articular TNF-α blockade therapy. Analysis of IL-1β, IL-1Ra, IL-6 and IL-22 concentrations in the SF post-treatment showed a statistically significant decrease with respect to basal values, suggesting these markers may provide reliable indications of response to TNF-α blockers in PsA patients [26].

Collins et al. purposefully mined the synovial tissue proteome in search of both predictive and pharmacodynamic markers of response to Etanercept- and Adalimumab-based anti-TNF-α therapy with the goal of minimizing the time patients spent on ineffective and costly treatments and avoiding unnecessary adverse effects [27]. Analysis of synovial tissue obtained from patients at both pre-treatment and 3 months post-treatment with Etanercept led to the identification of 21 predictive protein candidates indicative of patients who were at a higher risk of an adverse clinical outcome. Additionally, using synovial tissue at baseline from patients who either responded or did not respond to Etanercept treatment revealed eight potential pharmacodynamic biomarkers including serum albumin (ALB), collagen alpha 3 (COL4A3), annexin A1 (ANXA1) and A2 (ANXA2), Ig kappa chain C, BTB/POZ domain-containing protein, and tryptase. Similar analyses conducted on patients receiving Adalimumab treatment identified 26 predictive biomarker candidates and 14 pharmacodynamic biomarker candidates. In both treatment cohorts, ALB, COL4A3, ANXA1, and ANXA2 consistently demonstrated altered protein expression between those that did and did not respond well to the anti-TNF-α therapy, thus implying strong biomarker potential for these proteins in differentiating patients likely to benefit from the treatment. The authors acknowledged that the acquisition of synovial tissue samples is an extremely invasive process and, therefore, unlikely to be used for routine diagnostic purposes. Validation in more accessible biological fluids, such as serum, is necessary to ensure the clinical utility of these protein candidates.

Likewise, Ademowo et al. sought to identify a multi-parametric biomarker panel for the prediction of response to treatment according to protein profiles generated from the synovial tissue proteome [30]. Patient biopsy samples were acquired at baseline and 4 weeks following treatment with Adalimumab or placebo. Responder and non-responder protein profiles (*n* = 10) were generated using label-free, liquid chromatography MS of which 107 proteins were considered for the potential biomarker panel. Confirmation and pre-validation of the final 57 protein-based biomarker panel was completed with targeted proteomic analysis using multiple reaction monitoring (MRM) assays. Random forest analysis of the biomarker panels’ ability to predict patient response to Adalimumab resulted in an area under the curve (AUC) of 0.76. Although modest, pre-validation in a blinded independent cohort showed encouraging results with accurate predictions of patients’ response categories for six of the seven patients tested. For the ultimate translation of these findings into a diagnostic kit, it is valuable to validate in less invasive biological fluids with a much larger sample size. Furthermore, the use of such a kit in clinical practice would greatly benefit from a reduced number of biomarkers in the panel to ensure both timely and accurate disease management.

With the help of label-free, liquid chromatography tandem MS, Cretu et al. mined the PsA SF proteome for novel protein mediators of PsA [31]. Biomarker discovery resulted in the detection of 44 significantly upregulated proteins in PsA SF, of which 17 were determined to be strong biomarker candidates. Significant proteins identified include alpha-defensin 1 (DEFA1), histone 4 (H4) and histone 2A type I A (HIST1H2AA), all of which have demonstrated properties of antimicrobial activity. Observations of dysbiosis in the gut microbiota of PsA patients have been associated with possible dissemination of systemic inflammation [32]. The detection of significantly upregulated proteins in PsA SF with antimicrobial properties corroborates with these findings and warrants a further examination into the possible association between microbiome changes in PsA patients and the onset of joint pathology. Of the 17 overexpressed biomarker candidates, 12 proteins were verified to be significantly upregulated in a second cohort of PsA SF samples. A subset of these markers, including MMP-3, CD5L and MPO, was further found to be overexpressed in PsA serum compared to PsC serum thereby suggesting a strong clinical potential for these proteins to serve as diagnostic biomarkers of PsA [20].

## 4. Alternative Biomarker Sources

Urine is an entirely non-invasive source of proteomic biomarkers. Several advantages facilitate the use of urine as a bodily fluid for diagnostic purposes, including the ease and frequency of sample collection, the availability of large volumes, its low dynamic range and high stability due to the absence of high abundance serum proteins and proteolytic agents, respectively [33].

In the context of PsA, urinary biomarkers may serve as novel classifiers for the differential diagnosis of inflammatory arthritides among which there is a lack of highly sensitive and specific diagnostic tests. Siebert et al. attempted to address this clinical need by examining the proteomes of fifty RA, PsA, osteoarthritis (OA), or inflammatory bowel disease (IBD) patients and fifty healthy controls [34]. Using a capillary electrophoresis system coupled online to a MicroTOF (time-of-flight) mass spectrometer, they identified up to 566 peptides (RA) with 170 peptides being significantly associated with PsA compared to the other disease groups. The classifiers were established according to the top 45 significantly associated proteins and subsequently validated on a blinded test set of urine samples. The resulting receiver operating characteristics (ROC) curve for the performance of the PsA classifier had an AUC of 0.97, demonstrating high accuracy of disease detection. Their findings become especially important in primary care settings where the overlapping pathophysiologies of RA and PsA make them increasingly difficult to distinguish between. Although promising, the authors acknowledge the need for large-scale validation among an independent cohort of urine samples.

The nature in which PsA primarily develops following the onset of PsC led Cretu et al. to investigate the PsA skin proteome in efforts to catch undiagnosed PsA in PsC patients [28]. Using high-throughput, label-free mass spectrometry, they generated a list of 47 candidate proteins significantly elevated in lesioned PsA skin. They proceeded to verify these candidates using selected reaction monitoring (SRM) assays in the discovery sample set and a new set of skin biopsies. Subsequent small-scale ELISA validation of two verified candidates: β_5_ integrin (ITGB5) and periostin (POSTN), demonstrated significant elevation in PsA serum. Both ITGB5 and POSTN have been implicated in inflammatory signaling pathways [35,36]; however, this was the first time they were described in the context of PsA. By delineating the mechanistic roles ITGB5 and POSTN play in aberrant joint processes, we may advance our understanding of potentially key drivers of PsA.

## 5. Future Perspectives

There remains a large unmet need for sensitive diagnosis, prognosis and treatment of PsA in PsC patients. Proteomic studies provide numerous opportunities for the identification of soluble biomarkers which may elucidate the molecular mechanisms driving PsA pathogenesis, as well as serve as novel screening measures and therapeutic targets. Despite numerous proteomic studies, validation of protein candidates in a larger cohort is urgently needed to ensure potential biomarkers proceed down the biomarker pipeline to clinical use. Integration with other -omic domains, such as genomic or metabolomic characterization, will highlight aberrant signaling pathways likely driving the onset of psoriatic disease. In combination with emerging technologies, proteomic profiling will open novel avenues for disease detection and management, while continuing to broaden our understanding of the forces driving joint pathologies.

## Figures and Tables

**Table 1:** Candidate psoriatic arthritis (PsA) protein biomarkers and their probable role in clinical practice.

Putative Marker	Biological Source	Biomarker Type	Purpose
S100A8/S100A9	Serum	Prognostic	Differentiate between mild and severe forms of PsA [15]
IL-6	Serum	Monitoring	Disease activity measure [17]
CXCL10	Serum	Prognostic	Predict the development of PsA in PsC patients [18]
CD5L, ITGB5, M2BP, MPO, MMP3, CRP	Serum	Diagnostic	Detect the presence of PsA [20]
Anti-MCV	Serum	Prognostic	Differentiate between mild and severe forms of PsA [21]
MMP3, MIA	Serum	Predictive	Response to TNFi therapy [22,23]
SERPINA11, CFHR5, CRTAC1, F9	Serum	Prognostic	Differentiate between the onset of RA and PsA [25]
IL-1β, IL-1Ra, IL-6, IL-22	SF	Pharmacodynamic	Degree of response to TNFi treatment [26]
ALB, COL4A3, ANXA1, ANXA2	Synovial tissue	Predictive	Response to anti-TNF-α therapy [27]
ITGB5, POSTN	Lesional skin biopsy	Diagnostic	Detect the presence of PsA [28]

## References

[1] Nestle F.O., Kaplan D.H., Barker J. (2009). Psoriasis. N. Engl. J. Med..

[2] Ritchlin C.T., Colbert R.A., Gladman D.D. (2017). Psoriatic arthritis. N. Engl. J. Med..

[3] Chandran V., Schentag C.T., Brockbank J.E., Pellett F.J., Shanmugarajah S., Toloza S.M.A., Rahman P., Gladman D.D. (2009). Familial aggregation of psoriatic arthritis. Ann. Rheum. Dis..

[4] FitzGerald O., Haroon M., Giles J.T., Winchester R. (2015). Concepts of pathogenesis in psoriatic arthritis: Genotype determines clinical phenotype. Arthritis Res. Ther..

[5] Gladman D.D., Anhorn K.A., Schachter R.K., Mervart H. (1986). Hla antigens in psoriatic arthritis. J. Rheumatol..

[6] Eder L., Chandran V., Pellet F., Shanmugarajah S., Rosen C.F., Bull S.B., Gladman D.D. (2012). Human leucocyte antigen risk alleles for psoriatic arthritis among patients with psoriasis. Ann. Rheum. Dis..

[7] Stuart P.E., Nair R.P., Tsoi L.C., Tejasvi T., Das S., Kang H.M., Ellinghaus E., Chandran V., Callis-Duffin K., Ike R. (2015). Genome-wide association analysis of psoriatic arthritis and cutaneous psoriasis reveals differences in their genetic architecture. Am. J. Hum. Genet..

[8] Tang F., Sally B., Ciszewski C., Abadie V., Curran S.A., Groh V., FitzGerald O., Winchester R.J., Jabri B. (2013). Interleukin 15 primes natural killer cells to kill via nkg2d and cpla2 and this pathway is active in psoriatic arthritis. PLoS ONE.

[9] Mameli A., Cauli A., Taccari E., Scarpa R., Punzi L., Lapadula G., Peluso R., Ramonda R., Spadaro A., Iannone F. (2008). Association of mica alleles with psoriatic arthritis and its clinical forms. A multicenter italian study. Clin. Exp. Rheumatol..

[10] Tillett W., Costa L., Jadon D., Wallis D., Cavill C., McHugh J., Korendowych E., McHugh N. (2012). The classification for psoriatic arthritis (caspar) criteria—A retrospective feasibility, sensitivity, and specificity study. J. Rheumatol..

[11] Biomarkers Definitions Working Group (2001). Biomarkers and surrogate endpoints: Preferred definitions and conceptual framework. Clin. Pharmacol. Ther..

[12] Mc Ardle A., Flatley B., Pennington S.R., FitzGerald O. (2015). Early biomarkers of joint damage in rheumatoid and psoriatic arthritis. Arthritis Res. Ther..

[13] Mayeux R. (2004). Biomarkers: Potential uses and limitations. NeuroRx.

[14] Chandramouli K., Qian P.-Y. (2009). Proteomics: Challenges, techniques and possibilities to overcome biological sample complexity. Hum. Genom. Proteom. HGP.

[15] Hansson C., Eriksson C., Alenius G.-M. (2014). S-calprotectin (s100a8/s100a9): A potential marker of inflammation in patients with psoriatic arthritis. J. Immunol. Res..

[16] Scrivo R., Conigliaro P., Riccieri V., Di Franco M., Alessandri C., Spadaro A., Perricone R., Valesini G. (2015). Distribution of interleukin-10 family cytokines in serum and synovial fluid of patients with inflammatory arthritis reveals different contribution to systemic and joint inflammation. Clin. Exp. Immunol..

[17] Alenius G.M., Eriksson C., Rantapaa Dahlqvist S. (2009). Interleukin-6 and soluble interleukin-2 receptor alpha-markers of inflammation in patients with psoriatic arthritis?. Clin. Exp. Rheumatol..

[18] Abji F., Pollock R.A., Liang K., Chandran V., Gladman D.D. (2016). Brief report: Cxcl10 is a possible biomarker for the development of psoriatic arthritis among patients with psoriasis. Arthritis Rheumatol..

[19] Chandran V., Cook R.J., Edwin J., Shen H., Pellett F.J., Shanmugarajah S., Rosen C.F., Gladman D.D. (2010). Soluble biomarkers differentiate patients with psoriatic arthritis from those with psoriasis without arthritis. Rheumatology.

[20] Cretu D., Gao L., Liang K., Soosaipillai A., Diamandis E.P., Chandran V. (2017). Novel serum biomarkers differentiate psoriatic arthritis from psoriasis without psoriatic arthritis. Arthritis Care Res..

[21] Dalmady S., Kiss M., Kepiro L., Kovacs L., Sonkodi G., Kemeny L., Gyulai R. (2013). Higher levels of autoantibodies targeting mutated citrullinated vimentin in patients with psoriatic arthritis than in patients with psoriasis vulgaris. Clin. Dev. Immunol..

[22] Van Kuijk A.W.R., DeGroot J., Koeman R.C., Sakkee N., Baeten D.L., Gerlag D.M., Tak P.P. (2010). Soluble biomarkers of cartilage and bone metabolism in early proof of concept trials in psoriatic arthritis: Effects of adalimumab versus placebo. PLoS ONE.

[23] Chandran V., Shen H., Pollock R.A., Pellett F.J., Carty A., Cook R.J., Gladman D.D. (2013). Soluble biomarkers associated with response to treatment with tumor necrosis factor inhibitors in psoriatic arthritis. J. Rheumatol..

[24] Cauza E., Hanusch-Enserer U., Frischmuth K., Fabian B., Dunky A., Kostner K. (2006). Short-term infliximab therapy improves symptoms of psoriatic arthritis and decreases concentrations of cartilage oligomeric matrix protein. J. Clin. Pharm. Ther..

[25] Grazio S., Razdorov G., Erjavec I., Grubisic F., Kusic Z., Punda M., Anticevic D., Vukicevic S., Grgurevic L. (2013). Differential expression of proteins with heparin affinity in patients with rheumatoid and psoriatic arthritis: A preliminary study. Clin. Exp. Rheumatol..

[26] Fiocco U., Sfriso P., Oliviero F., Roux-Lombard P., Scagliori E., Cozzi L., Lunardi F., Calabrese F., Vezzu M., Dainese S. (2010). Synovial effusion and synovial fluid biomarkers in psoriatic arthritis to assess intraarticular tumor necrosis factor-alpha blockade in the knee joint. Arthritis Res. Ther..

[27] Collins E.S., Butt A.Q., Gibson D.S., Dunn M.J., Fearon U., van Kuijk A.W., Gerlag D.M., Pontifex E., Veale D.J., Tak P.P. (2016). A clinically based protein discovery strategy to identify potential biomarkers of response to anti-tnf-alpha treatment of psoriatic arthritis. Proteom. Clin. Appl..

[28] Cretu D., Liang K., Saraon P., Batruch I., Diamandis E.P., Chandran V. (2015). Quantitative tandem mass-spectrometry of skin tissue reveals putative psoriatic arthritis biomarkers. Clin. Proteom..

[29] Guggino G., Ciccia F., Di Liberto D., Lo Pizzo M., Ruscitti P., Cipriani P., Ferrante A., Sireci G., Dieli F., Fournie J.J. (2016). Interleukin (il)-9/il-9r axis drives gammadelta t cells activation in psoriatic arthritis patients. Clin. Exp. Immunol..

[30] Ademowo O.S., Hernandez B., Collins E., Rooney C., Fearon U., van Kuijk A.W., Tak P.-P., Gerlag D.M., FitzGerald O., Pennington S.R. (2016). Discovery and confirmation of a protein biomarker panel with potential to predict response to biological therapy in psoriatic arthritis. Ann. Rheum. Dis..

[31] Cretu D., Prassas I., Saraon P., Batruch I., Gandhi R., Diamandis E.P., Chandran V. (2014). Identification of psoriatic arthritis mediators in synovial fluid by quantitative mass spectrometry. Clin. Proteom..

[32] Scher J.U., Ubeda C., Artacho A., Attur M., Isaac S., Reddy S.M., Marmon S., Neimann A., Brusca S., Patel T. (2015). Decreased bacterial diversity characterizes the altered gut microbiota in patients with psoriatic arthritis, resembling dysbiosis in inflammatory bowel disease. Arthritis Rheumatol..

[33] Adachi J., Kumar C., Zhang Y., Olsen J.V., Mann M. (2006). The human urinary proteome contains more than 1500 proteins, including a large proportion of membrane proteins. Genome Biol..

[34] Siebert S., Porter D., Paterson C., Hampson R., Gaya D., Latosinska A., Mischak H., Schanstra J., Mullen W., McInnes I. (2017). Urinary proteomics can define distinct diagnostic inflammatory arthritis subgroups. Sci. Rep..

[35] Nakajima M., Honda T., Miyauchi S., Yamazaki K. (2014). Th2 cytokines efficiently stimulate periostin production in gingival fibroblasts but periostin does not induce an inflammatory response in gingival epithelial cells. Arch. Oral Boil..

[36] Lin J., Zhou Z., Huo R., Xiao L., Ouyang G., Wang L., Sun Y., Shen B., Li D., Li N. (2012). Cyr61 induces il-6 production by fibroblast-like synoviocytes promoting th17 differentiation in rheumatoid arthritis. J. Immunol..

